# Supplementary material for: Assessment of software methods for estimating protein-protein relative binding affinities
Source: PLoS One. 2020 Dec 21;15(12):e0240573. doi: 10.1371/journal.pone.0240573 (PMC7751979; doi:10.1371/journal.pone.0240573)
Supplement: S1 File — (DOCX) [file pone.0240573.s001.docx]

**Assessment of Software Methods for Estimating Protein-Protein Relative Binding Affinities**

Tawny R. Gonzalez^1¶^, Kyle P. Martin^1,2¶^, Jonathan E. Barnes^1,2^, Jagdish Suresh Patel^1,3*^, F. Marty Ytreberg^1,2*^

^1^Institute for Modeling Collaboration and Innovation, University of Idaho, Moscow, Idaho, United States of America

^2^Department of Physics, University of Idaho, Moscow, Idaho, United States of America

^3^Department of Biological Sciences, University of Idaho, Moscow, Idaho, United States of America

* Corresponding authors

E-mail: thejagdishpatel@gmail.com (JSP)

E-mail: ytreberg@uidaho.edu (FMY)

^¶^These authors contributed equally to this work.

**Methods**

**Input structure file preparation**

The 3-D structures of 16 protein-protein complexes were obtained from the PDB server (<https://www.rcsb.org>). Each structure file was edited to preserve only the coordinates of the number of interacting chains listed in the SKEMPI database [22]. All missing residues and atoms were then added using MODELLER software [57]. For cases where a method required structure files for mutant as well as wildtype complexes, *BuildModel* command from FoldX software [13, 39] was used to generate mutant structure files for all the experimental mutations in this study. In cases where a method could only analyze two chains from a given protein complex, multiple chains from each partnering protein such as heavy and light chains of antibody or multiple chains of homomeric protein were combined prior to using them as an input.

###### **BindProfX and BindProfX+FoldX**

BindProfX [15, 16] calculates binding affinity by calculating a profile conservation score based on homologous binding interfaces. BindProfX online web server (<https://zhanglab.ccmb.med.umich.edu/BindProfX/>) was used to calculate ∆∆*G* (relative binding affinity) values. An additional option is available under the advanced options section in which BindProfX calculated ∆∆*G* values can be combined with values estimated by FoldX software. To estimate ∆∆*G* values, the wildtype structure file was uploaded along with a list containing the experimental mutations for each complex. BindProfX focuses on the conservation of binding interfaces as a way to predict binding affinity hence focuses only on the interface mutations. As such, some experimental mutation occurring far from the interface had to be excluded from the ∆∆*G* calculation. The total number of excluded mutations across all protein complexes was 36. ∆∆*G* values were pulled directly from the final output for each submission.

###### **iSEE**

The iSEE software [41] compiles a file containing amino acid positions and a variety of energy features to calculate ∆∆*G* values. Positional information (PSSM) was generated by PSIBLAST server (<https://www.ebi.ac.uk/Tools/sss/psiblast/>) by providing a FASTA sequence of a wildtype protein complex as an input. Energy features such as van der Waals and desolvation energies were calculated using the HADDOCK 2.2 web server (<http://haddock.science.uu.nl/services/HADDOCK2.2/haddockserver-refinement.html>) [53-55] for each wildtype and mutant complex. An in-house script was used to automate the upload process of all complexes. These features were compiled into a single file containing both wildtype and mutant data for each protein complex and analyzed using the R script available for download from <https://github.com/haddocking/iSee>. ∆∆*G* value for each mutant was then obtained from the output file.

###### **DCOMPLEX**

DCOMPLEX [14] is a method adapted from the DFIRE-based all-atom statistical potential developed for analyzing folding stability. DCOMPLEX online web server is no longer available, but the standalone program written in C++ is available for download at the following site: <http://sparks-lab.org/Publications> (or download by clicking on this link: <https://sparks-lab.org/download/dcomplex2.tar.gz>). Minor edits were needed in the downloaded code to make it compatible with a new version of C++ compiler and to accommodate the size of some of the protein complexes. This method calculates ∆*G* value hence the ∆*G* for wildtype and mutant complexes were first calculated individually and then used to estimate ∆∆*G* values for each mutation.

###### **JayZ and EasyE**

JayZ and EasyE [40, 42] are distinct binding affinity predictors. Both methods are based on Rosetta software [43] and available as Python script (<https://sourcesup.renater.fr/frs/?group_id=3441>). These methods deviate in workflow after the initial calculation of energy matrices. EasyE generates binding affinity using an internal energy approximation calculated from the global minimum energy. JayZ utilizes partition function approximations based on conformational ensembles. Both the methods allow users to specify a list of mutations that the method is then able to calculate in one setting. This list can be optimized based on the available hardware to achieve efficiency. Default command options were used for both JayZ and EasyE to calculate ∆*G* value, which were then used to calculate ∆∆*G* value for each mutation.

###### **FoldX and MD+FoldX Snapshots**

FoldX software [13, 39] uses a semi-empirical method for calculating ΔΔ*G* values. FoldX calculations were carried out using a single experimental structure (FoldX) and 100 snapshots extracted from 100 ns long molecular dynamics (MD) simulations (MD+FoldX) of all 16 wildtype protein complexes. ΔΔ*G* values for all the mutations from 16 protein complexes were calculated using the same FoldX and MD+FoldX protocols reported in our previous studies [45, 46].
