# Supplementary figures and images for: Assessment of software methods for estimating protein-protein relative binding affinities
Source: PLoS One. 2020 Dec 21;15(12):e0240573. doi: 10.1371/journal.pone.0240573 (PMC7751979; doi:10.1371/journal.pone.0240573)

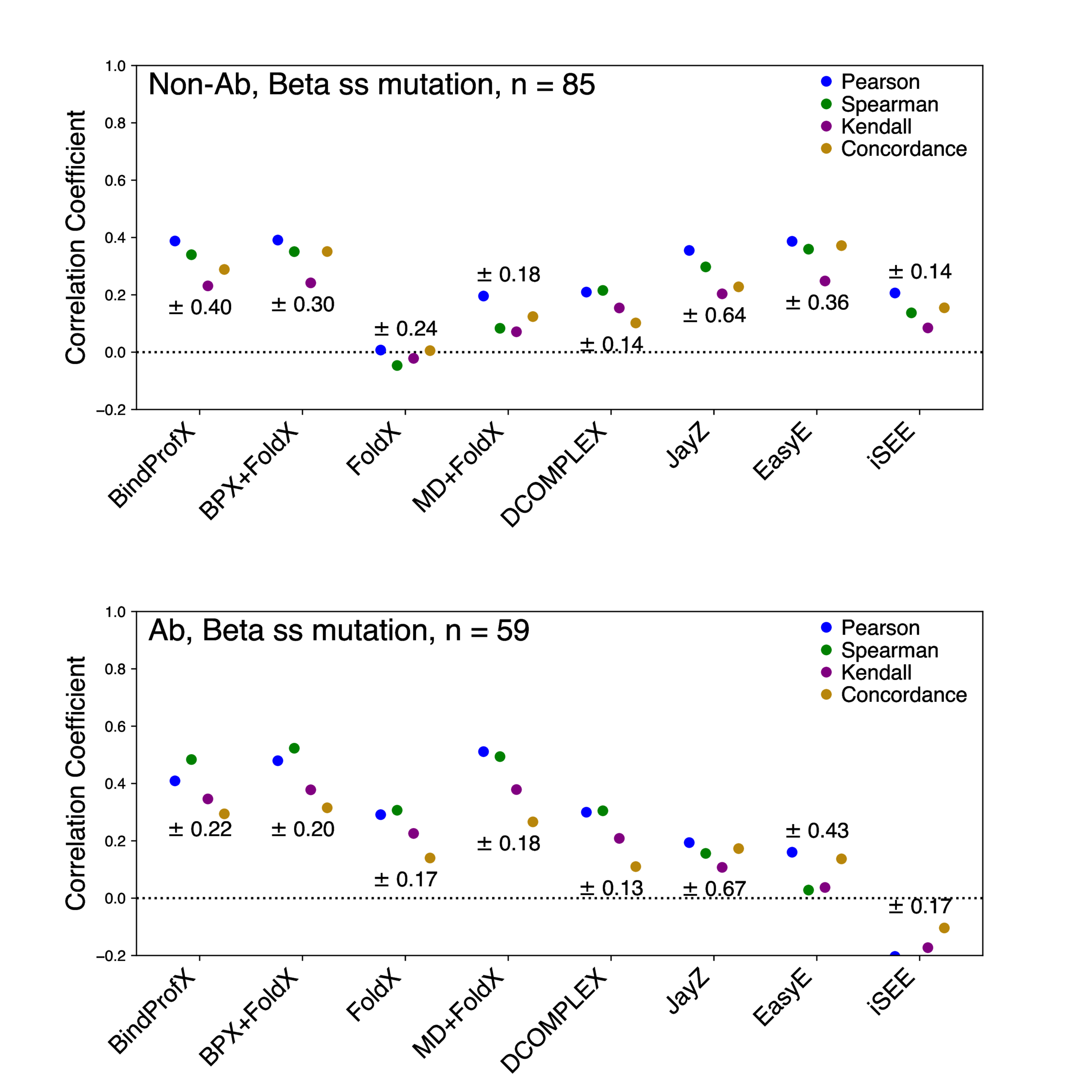

Supplement: S1 Fig — The error for each method is reported under the correlation points. (TIFF) [file pone.0240573.s004.tiff]

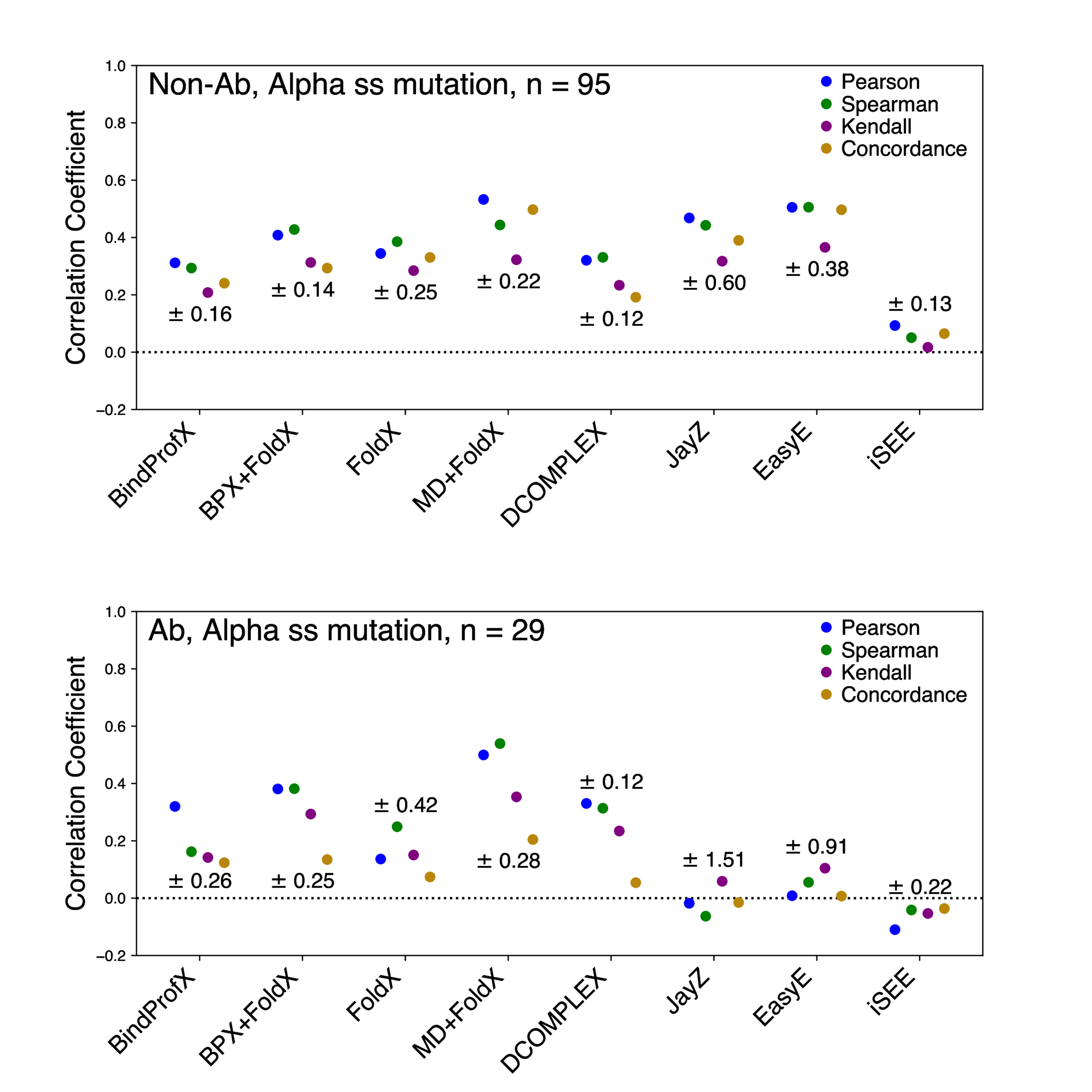

Supplement: S2 Fig — The error for each method is reported under the correlation points. (TIFF) [file pone.0240573.s005.tiff]

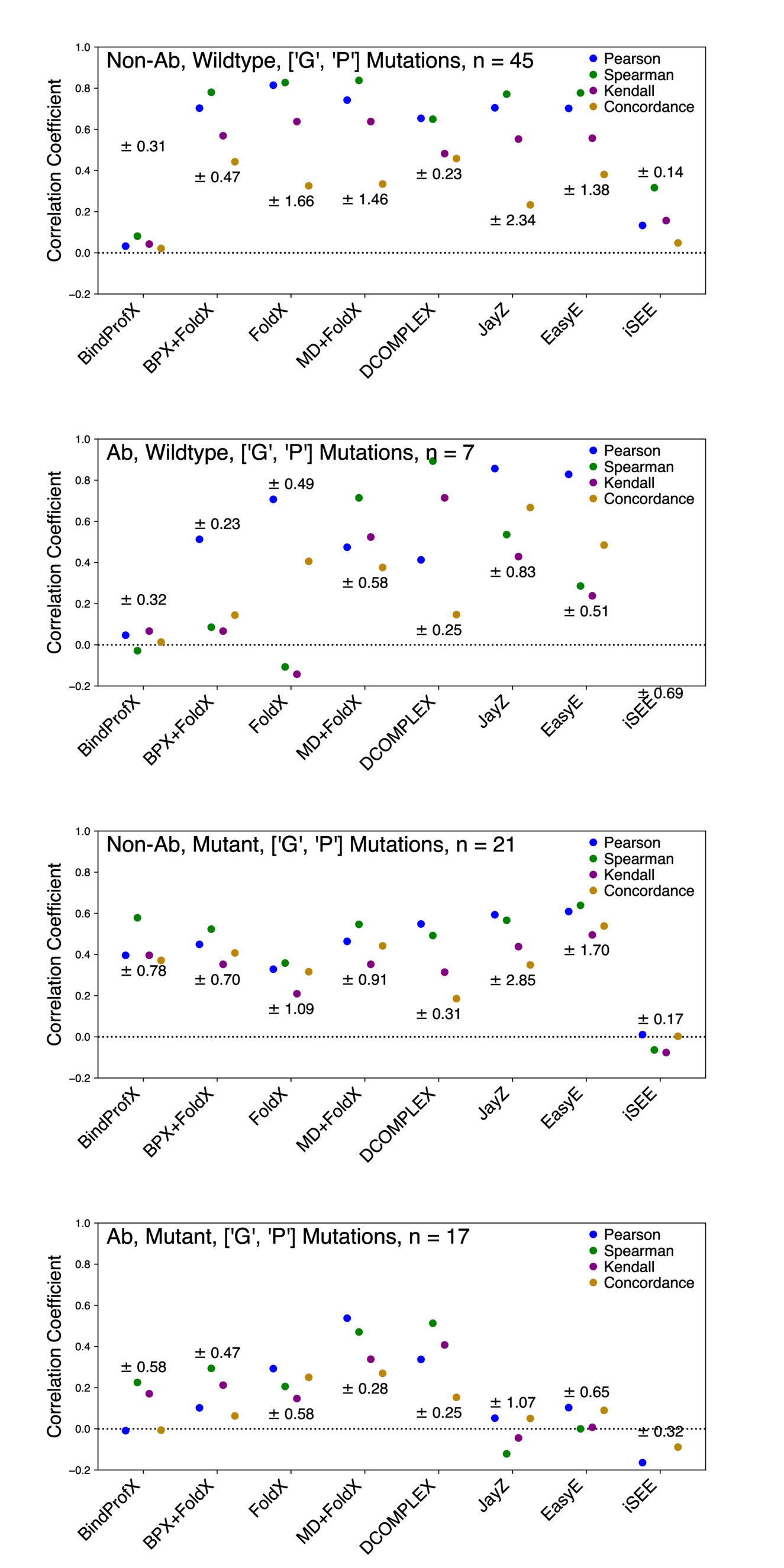

Supplement: S3 Fig — The error for each method is reported under the correlation points. (TIFF) [file pone.0240573.s006.tiff]

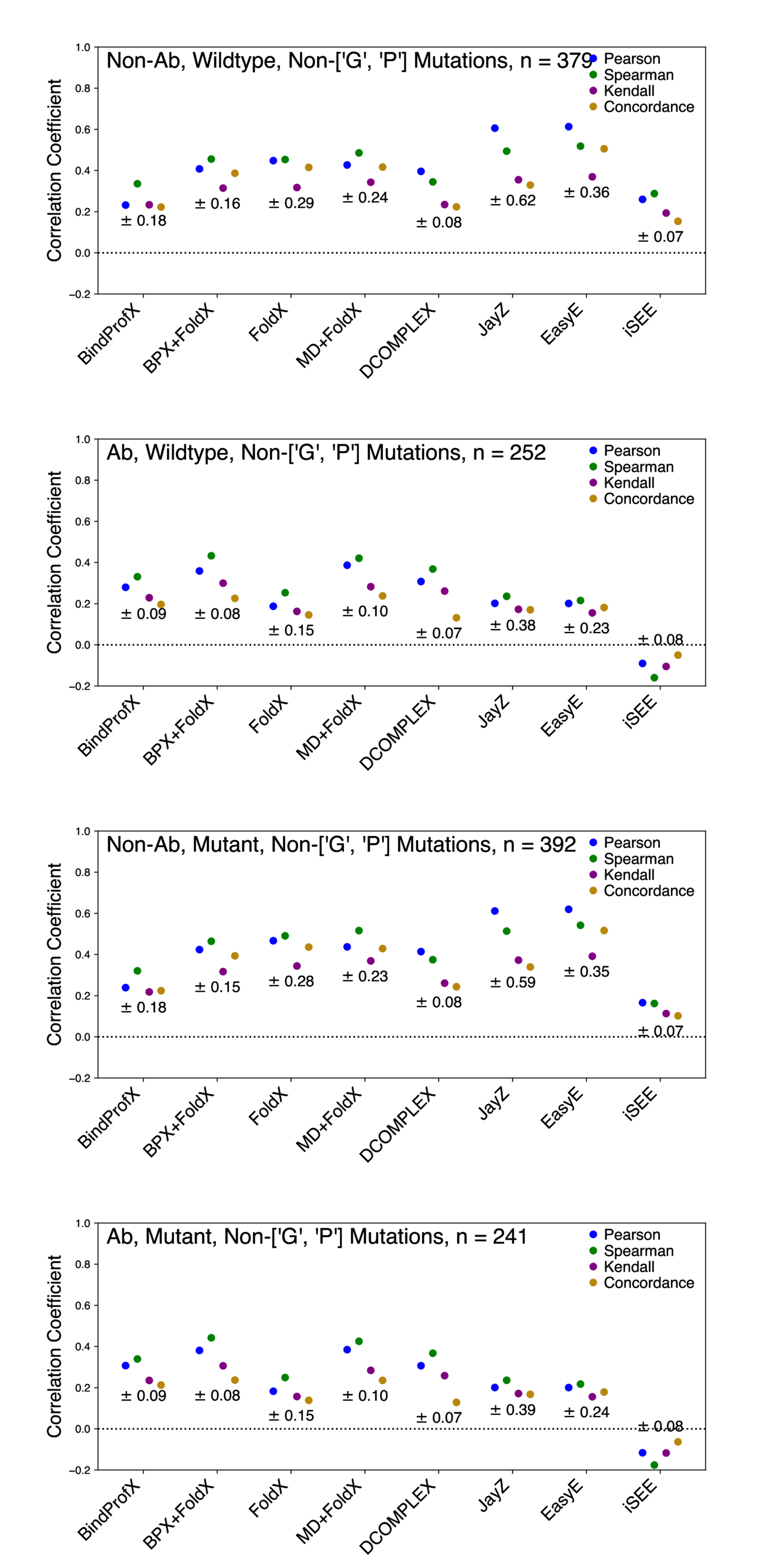

Supplement: S4 Fig — The error for each method is reported under the correlation points. (TIFF) [file pone.0240573.s007.tiff]
